# Berteroin Present in Cruciferous Vegetables Exerts Potent Anti-Inflammatory Properties in Murine Macrophages and Mouse Skin

**DOI:** 10.3390/ijms151120686

**Published:** 2014-11-11

**Authors:** Yoo Jin Jung, Jae In Jung, Han Jin Cho, Myung-Sook Choi, Mi-Kyung Sung, Rina Yu, Young-Hee Kang, Jung Han Yoon Park

**Affiliations:** 1 Department of Food Science and Nutrition, Hallym University, Chuncheon 200-702, Korea; E-Mails: betyloving@naver.com (Y.J.J.); wjdwodls@hallym.ac.kr (J.I.J.); yhkang@hallym.ac.kr (Y.-H.K.); 2 WCU Biomodulation Major, Department of Agricultural Biotechnology and Center for Food and Bioconvergence, Seoul National University, Seoul 151-921, Korea; E-Mail: hanjini@snu.ac.kr; 3 Center for Food and Nutritional Genomics Research and Department of Food Science and Nutrition, Kyungpook National University, Daegu 702-701, Korea; E-Mail: mschoi@knu.ac.kr; 4 Department of Food and Nutrition, Sookmyung Women’s University, Seoul 140-742, Korea; E-Mail: mksung@sookmyung.ac.kr; 5 Department of Food Science and Nutrition, University of Ulsan, Ulsan 680-749, Korea; E-Mail: rinayu@ulsan.ac.kr; 6 Advanced Institutes of Convergence Technology, Seoul National University, Suwon, Gyonggi-do 443-270, Korea

**Keywords:** inflammation, berteroin, iNOS, COX-2

## Abstract

Berteroin (5-methylthiopentyl isothiocyanate) is a sulforaphane analog present in cruciferous vegetables, including Chinese cabbage, rucola salad leaves, and mustard oil. We examined whether berteroin exerts anti-inflammatory activities using lipopolysaccharide (LPS)-stimulated Raw 264.7 macrophages and 12-O-tetradecanoylphorbol-13-acetate (TPA)-induced mouse skin inflammation models. Berteroin decreased LPS-induced release of inflammatory mediators and pro-inflammatory cytokines in Raw 264.7 macrophages. Berteroin inhibited LPS-induced degradation of inhibitor of κBα (IκBα) and nuclear factor-κB p65 translocation to the nucleus and DNA binding activity. Furthermore, berteroin suppressed degradation of IL-1 receptor-associated kinase and phosphorylation of transforming growth factor β activated kinase-1. Berteroin also inhibited LPS-induced phosphorylation of p38 MAPK, ERK1/2, and AKT. In the mouse ear, berteroin effectively suppressed TPA-induced edema formation and down-regulated iNOS and COX-2 expression as well as phosphorylation of AKT and ERK1/2. These results demonstrate that berteroin exhibits potent anti-inflammatory properties and suggest that berteroin can be developed as a skin anti-inflammatory agent.

## 1. Introduction

Inflammation is the cause of a broad variety of physiological and pathological processes. Inflammation is one of the defense mechanisms against tissue injury caused by biological, chemical, and physical factors [1]. However, inflammation underlies the development of multiple diseases including cancer, diabetes, inflammatory bowel diseases, and cardiovascular diseases (reviewed in [2]). Inflammation is essentially mediated by pro-inflammatory mediators (e.g., nitric oxide (NO) and prostaglandin E_2_ (PGE_2_)) and pro-inflammatory cytokines, including tumor necrosis factor (TNF)-α, interleukin (IL)-6, and IL-1β [1,3]. The pro-inflammatory cytokines are associated with pathogenesis of carcinogenesis as well as tumor growth and metastasis [4].

NO is synthesized from L-arginine by NO synthase (NOS) that exists as three types: endothelial NOS (eNOS), neuronal NOS (nNOS) and inducible NOS (iNOS) [5]. Among these, nNOS and eNOS are always distributed in the intracellular region, whereas iNOS is expressed after exposure to lipopolysaccharide (LPS), interferon-γ, and various pro-inflammatory cytokines that contribute to the pathological mechanism of NO [6]. Normally, NO mediates the immune response that kills bacteria or eliminates tumor cells. However, hyper-production of NO causes tissue injury, genetic alterations, and neuronal damage by inducing dysregulated inflammation [7]. PGs synthesized by the action of cyclooxygenase (COX) play crucial roles in both acute and chronic skin inflammation induced by a variety of stimuli (reviewed in [8]). COX-1 is expressed in many tissues and organs and contributes to the maintenance of normal tissues, whereas COX-2 is induced by a number of stimuli. As dysregulated activation of COX-2 and iNOS play important roles in the development of several inflammatory diseases including cancer development [9], targeting iNOS and COX-2 in inflammatory cells is an efficient strategy for chemoprevention [10]. Additionally, aberrant overexpression and activity of COX-2 are involved in skin tumor promotion and progression (reviewed in [8]).

Toll-like receptors (TLRs) recognize specific molecular patterns that are present in microbial components, and the expression of inflammatory mediators and pro-inflammatory cytokines caused by LPS released by Gram-negative bacteria is largely controlled by the TLR4 signaling pathway (reviewed in [11]). When TLR4 is stimulated by LPS, the TLR4 downstream molecules (myeloid differentiation primary response gene 88 (MyD88), interlukin-1 receptor-associated kinases (IRAKs), and transforming growth factor β activated kinase-1 (TAK1)) are activated, and their signal transduction is mediated through distinct signaling cascades to activate nuclear factor (NF)-κB. NF-κB is activated by phosphorylation of IκB via activation of IκB kinase (IKK) and mitogen-activated protein kinases (MAPKs) such as p38 MAPK, extracellular signal-regulated kinases 1/2 (ERK1/2), and c-Jun N-terminal kinase (reviewed in [12,13,14]). NF-κB consists of p65 and p50 as a subunit-heterodimer, and is present in the cytosol as an inactive complex with inhibitor of κB (IκB). When IKK is activated by various pro-inflammatory stimuli such as LPS, IκB is phosphorylated and then degraded, resulting in the liberation of NF-κB [12,15]. The resulting free NF-κB is translocated to the nucleus where it regulates transcription of a broad variety of proteins involved in the process of inflammation [16].

**Figure 1:**
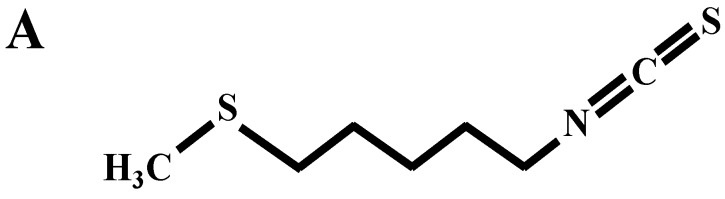

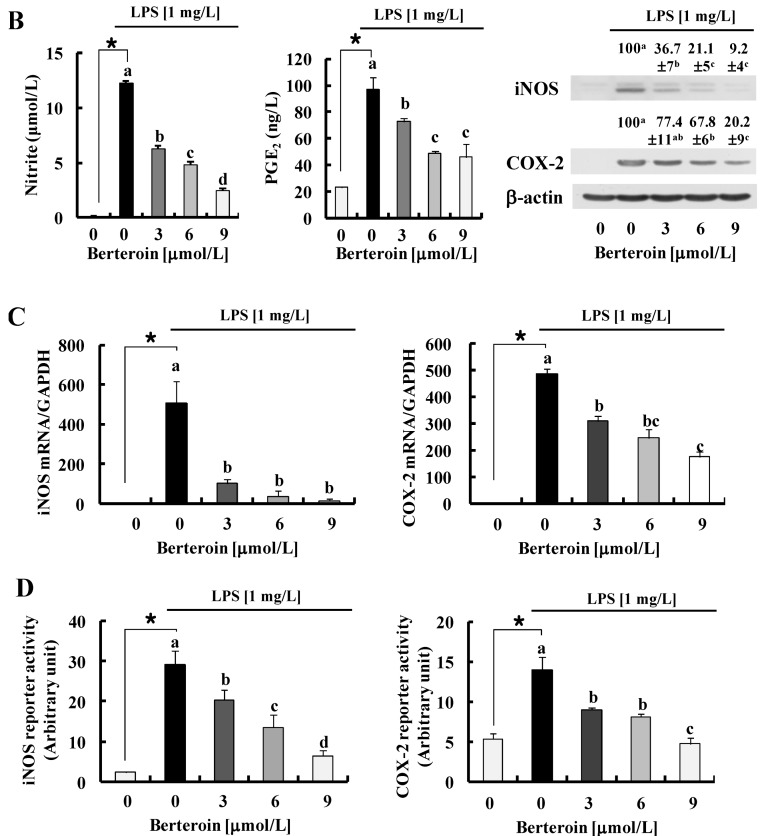
Berteroin inhibits nitric oxide (NO) and prostaglandin (PG)E_2_ production and decreases inducible nitric oxide synthase (iNOS) and cyclooxygenase-2 (COX)-2 expression in lipopolysaccharide (LPS)-stimulated Raw 264.7 cells. (**A**) The structure of berteroin is shown. (**B–D**) Raw 264.7 cells were plated in 24-well plates at 5 × 10^4^ cells/well in Dulbecco’s modified Eagle’s medium (DMEM) and 100 mL/L fetal bovine serum (FBS), serum-deprived in DMEM + 10 mL/L FBS, and treated for 24 h with various concentrations of berteroin in the absence or presence of 1 mg/L LPS; (**B**) Conditioned media were collected 24 h after berteroin treatment. The concentrations of NO and PGE_2_ in the conditioned media were measured using the Griess reagent system and a PGE_2_ enzyme linked immunosorbent assay (ELISA) kit, respectively. Western blotting was conducted using an anti-iNOS, COX-2, or β-actin antibodies with total cell lysates. Photographs of chemiluminescent detection of the blots, which were representative of three independent experiments, are shown. Densities were calculated by Image J software and shown above each blot; (**C**) Total RNA was isolated and reverse transcribed, and real-time polymerase chain reaction (PCR) was conducted. iNOS and COX-2 mRNAs were normalized to glyceraldehyde-3-phosphate dehydrogenase (GAPDH) mRNAs; (**D**) Cells were co-transfected with murine iNOS or COX-2 reporter gene construct and the *Renilla* control vector, and the transfected cells were plated in 24-well plates at 5 × 10^4^ cells/well. After serum deprivation, the cells were treated with the indicated concentrations of berteroin in the absence or presence of LPS for 6 h. Luciferase activity was analyzed by the dual-luciferase assay and normalized to *Renilla* luciferase activity. Results represent the means ± SEM from 3 independent experiments. * Significantly different from the untreated group (0 mg/L LPS + 0 μmol/L berteroin) (*p* < 0.05). Means with different letter (a, b, c, or d) are significantly different among the berteroin groups (*p* < 0.05).

Countless attempts have been made to identify substances from natural products that can inhibit inflammation without causing side effects. Among these products, isothiocyanates (ITCs) produced by myrosinase-mediated hydrolysis when cruciferous vegetables are chewed and/or chopped [17] possess chemopreventive activities (reviewed in [18]). ITCs are accountable for the chemopreventive effects imparted by high dietary intake of cruciferous vegetables. The anti-tumor effects of ITCs are mediated through a variety of interrelated pathways involved in detoxification, inflammation, and cell survival as well as epigenetic regulation (reviewed in [19]). Berteroin (5-methylthiopentyl isothiocyanate, see structure in Figure 1A) is a sulforaphane analogue with non-oxidized sulfur (–N=C=S) and exists in cruciferous vegetables including Chinese cabbage, rocket, and mustard oil [20]. Berteroin inhibits the growth of B16F10 melanoma cells *in vitro* and inhibits pulmonary colonization when 10 μmoles of berteroin is given orally 1 day after intravenous B16F10 injection [21]. However, the effect of berteroin on inflammation has not been investigated.

We have previously shown that phenethyl isothiocyanate (PITC), benzyl isothiocyanate (BITC), and erucin derived from cruciferous vegetables exhibit anti-inflammatory effects by inhibiting activation of ERK1/2, AKT, and NF-κB signaling in LPS-stimulated macrophages and 12-O-tetradecanoylphorbol-13-acetate (TPA)-induced inflammation in mouse skin [22,23,24]. In the present study, we examined whether berteroin exerts anti-inflammatory properties and assessed its underlying mechanisms using LPS-stimulated Raw 264.7 murine macrophages and a TPA-induced mouse ear edema model. Our results clearly show that berteroin effectively inhibited inflammatory responses in both macrophages and mouse skin.

## 2. Results and Discussion

### 2.1. Berteroin Inhibits the Production of NO, PGE_2_, and pro-Inflammatory Cytokines in LPS-Stimulated Raw 264.7 Cells

**Figure 2:**
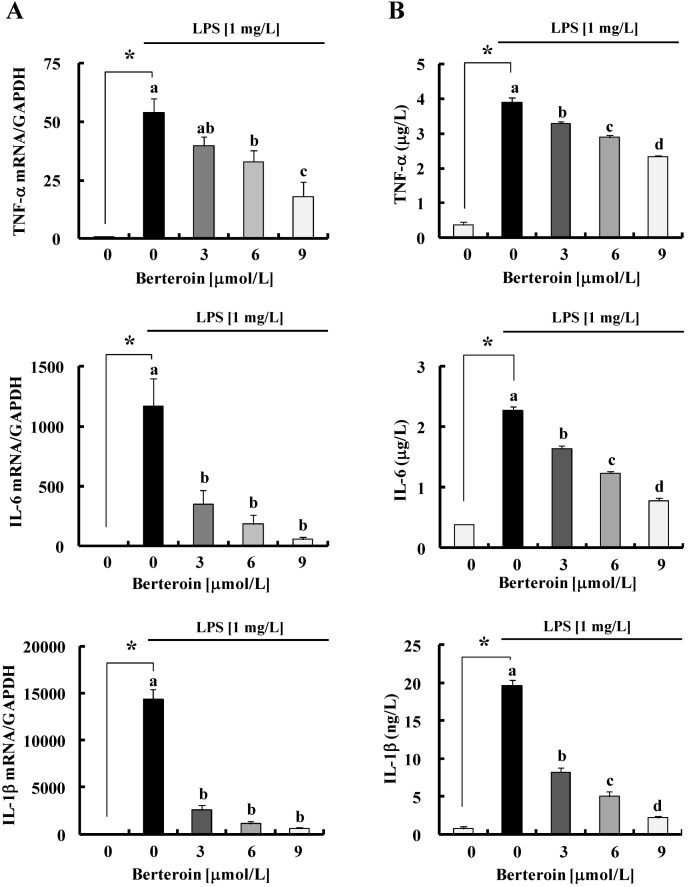
Berteroin decreases secretion and the transcripts of tumor necrosis factor (TNF)-α, interleukin (IL)-6, and IL-1β in lipopolysaccharide (LPS)-stimulated Raw 264.7 cells. The cells were treated with LPS and/or various concentrations of berteroin as described in Figure 1. (**A**) Total RNA was isolated and reverse-transcribed, and real-time PCR was performed. The mRNA levels of pro-inflammatory cytokines were normalized with those of GAPDH; (**B**) Conditioned media were collected after 24 h. The concentrations of TNF-α, IL-6, and IL-1β in the conditioned media were measured using the relevant ELISA kits. Each bar represents mean ± SEM (*n* = 4). * Significantly different from the untreated control group (0 mg/L LPS + 0 μmol/L berteroin) (*p* < 0.05). Means with different letter (a, b, c or d) are significantly different among the berteroin groups (*p* < 0.05).

To investigate whether berteroin inhibits the production of NO and PGE_2_ in macrophages, Raw 264.7 cells were treated with 3–9 μmol/L berteroin in the presence of LPS. Berteroin decreased the secretion of NO and PGE_2_ in LPS-stimulated Raw 264.7 cells in a dose-dependent manner (Figure 1B). These berteroin concentrations did not affect macrophage viability (data not shown). Therefore, we utilized these berteroin concentrations in subsequent experiments. Berteroin significantly decreased LPS-induced increases in the levels of iNOS and COX-2 protein and mRNAs estimated by Western blot and real-time PCR analyses, respectively (Figure 1B,C). Additionally, iNOS and COX-2 transcriptional activities estimated by reporter gene assays were dose-dependently inhibited by berteroin (Figure 1D). Similar to changes in iNOS and COX-2, the mRNA levels and secretion of TNF-α, IL-6, and IL-1β induced by LPS stimulation were significantly suppressed after treatment with berteroin (Figure 2A,B).

**Figure 3:**
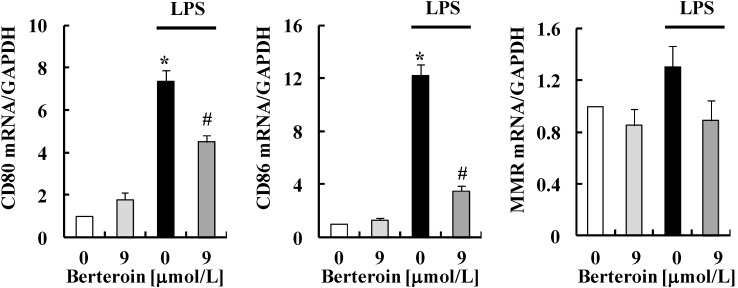
Berteroin decreases the transcripts of CD80 and CD86 in lipopolysaccharide (LPS)-stimulated Raw 264.7 cells. The cells were treated with 0 or 9 μmol/L berteroin and/or LPS as described in Figure 1. Total RNA was isolated and reverse-transcribed, and real-time PCR was performed. Each bar represents mean ± SEM (*n* = 3). * Significantly different from the untreated control group (0 mg/L LPS + 0 μmol/L berteroin) (*p* < 0.05). ^#^ Significantly different from the LPS control group (1 mg/L LPS + 0 μmol/L berteroin) (*p* < 0.05).

We next examined whether berteroin induces phenotype changes in Raw 264.7 cells. Real-time RT-PCR analysis showed that LPS markedly increased the mRNA levels of CD80 and CD86 (M1 macrophage markers), which was significantly suppressed by berteroin. However, neither LPS nor berteroin had any effect on the mRNA levels of macrophage mannose receptor (MMR, a M2 macrophage marker) in Raw 264.7 cells (Figure 3).

### 2.2. Berteroin Inhibits Activation of NF-κB Signaling in LPS-Stimulated Raw 264.7 Cells

**Figure 4:**
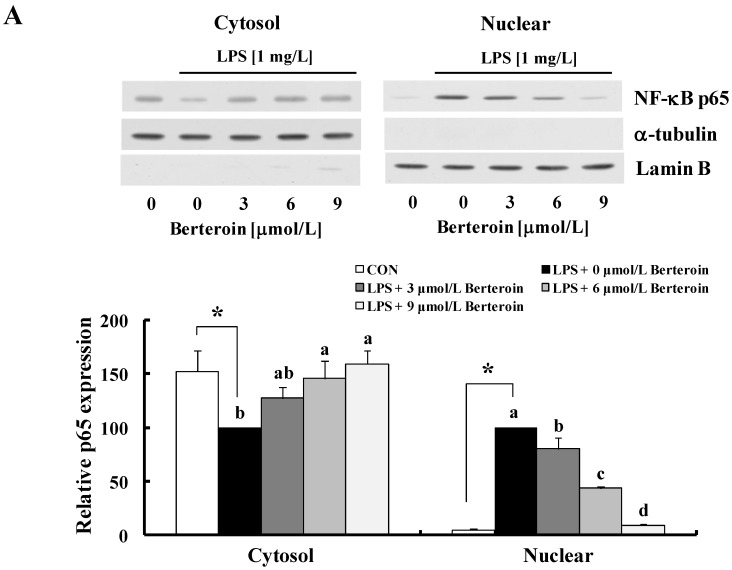

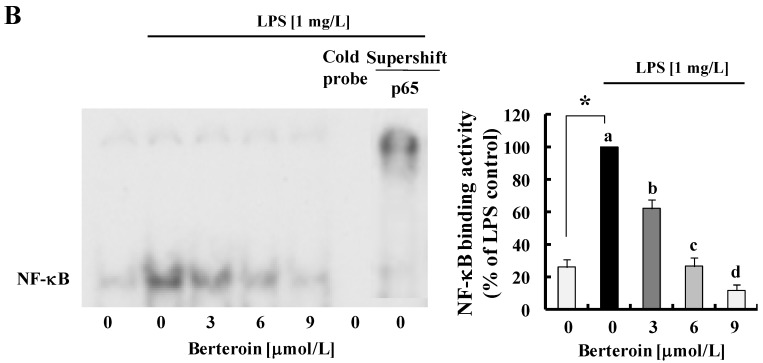
Berteroin inhibits nuclear factor (NF)-κB translocation to the nucleus and its DNA binding activity in lipopolysaccharide (LPS)-stimulated Raw 264.7 cells. The cells were treated with various concentrations of berteroin for 40 min. LPS was added, and the incubation was continued an additional 20 min. (**A**) Cytosolic and nuclear fractions were prepared 20 min after the incubation with LPS, and the levels of p65 were analyzed by Western blotting. Photographs of chemiluminescent detection of the blots, which were representative of three independent experiments, are shown (upper panel). Relative density (mean ± SEM, *n* = 3) was calculated using Image J software (lower panel). Levels of α-tubulin and lamin B were used as cytosolic and nuclear loading controls, respectively; (**B**) The effect of berteroin on the DNA binding activity of p65 was evaluated by electrophoretic mobility shift assay. The nuclear extracts were incubated with the [γ-^32^P]-labeled NF-κB consensus oligonucleotides for 30 min. Each sample was subjected to 5% nondenaturing gel electrophoresis. (Left) An autoradiograph of the dried gel, which was representative of three independent experiments, is shown. (Right) The relative abundance of each band was quantified, and the LPS control levels (1 mg/L LPS + 0 μmol/L berteroin) were set to 100%. Each bar represents the mean ± SEM (*n* = 3). * Significantly different from the control group (*p* < 0.05). Means with different letter (a, b, c or d) are significantly different among the berteroin groups (*p* < 0.05).

NF-κB is a key transcriptional regulator of pro-inflammatory gene expression. It induces transcription of a wide variety of genes including *COX-2*, *iNOS*, *IL-1*, and *TNF* genes [25] (reviewed in [26]). The Western blotting results revealed that LPS reduced the levels of cytosolic NF-κB p65 and increased those of nuclear p65, which were prevented by berteroin in a dose-dependent manner (Figure 4A). These results clearly indicate that berteroin inhibits p65 translocation to the nucleus. The electrophoretic mobility shift assay (EMSA) results revealed that LPS greatly increased NF-κB DNA binding activity, which was markedly suppressed in a berteroin dose-dependent manner (Figure 4B).

### 2.3. Berteroin Inhibits Degradation of IRAK1 and IκBα as Well as Phosphorylation of TAK1, IKKα/β, IκBα, AKT, and MAPKs in LPS-Stimulated Raw 264.7 Cells

**Figure 5:**
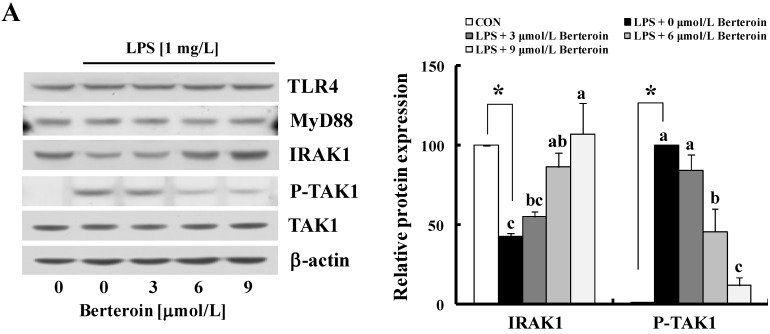

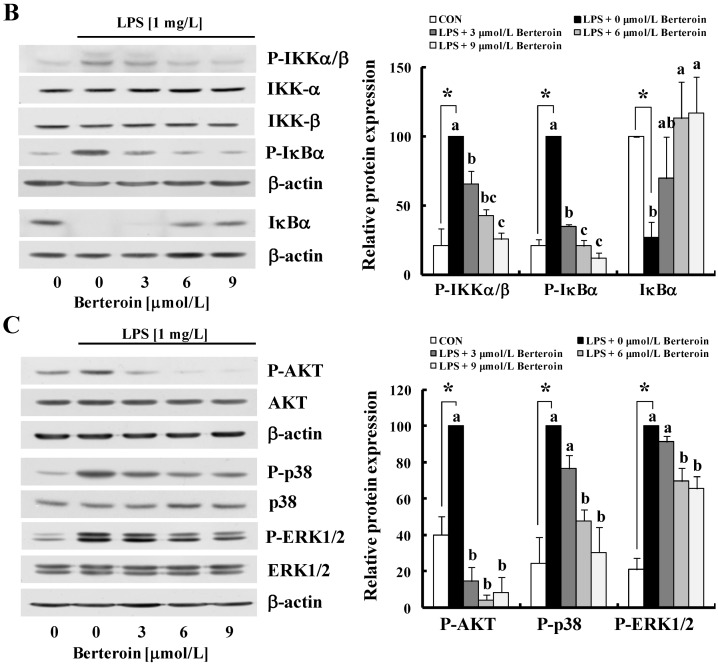
Berteroin inhibits phosphorylation of transforming growth factor β activated kinase-1 (TAK1), IκB kinase (IKK)α/β, IκBα, AKT, p38 mitogen activated protein kinase (MAPK), and extracellular regulated kinase (ERK)1/2 in lipopolysaccharide (LPS)-stimulated Raw 264.7 cells. The cells were treated with various concentrations of berteroin for 40 min. LPS was added and the incubation was continued for an additional 10 min (to determine IRAK1, TLR4, MyD88, P-TAK1, TAK1, P-IKKα/β, IKKα/β, P-IκBα, P-AKT, and AKT) or for 20 min (to determine IκBα, P-p38, p38, P-ERK1/2, and ERK1/2). Western blotting was conducted using total cell lysates (left panel). Densities were calculated using Image J software (right panel). Each bar represents mean ± SEM (*n* = 3). * Significantly different from the untreated group (*p* < 0.05). Means with different letter (a, b or c) are significantly different among the berteroin groups (*p* < 0.05).

It is generally known that LPS-mediated inflammation is regulated by the TLR4 signaling pathway (reviewed in [11]). Thus, we examined whether berteroin induces changes in the levels of TLR4, MyD88, TAK1, and IRAK1 in LPS-stimulated macrophages using Western blotting. The levels of TLR4, MyD88, and TAK1 remained unchanged by either LPS or berteroin treatment. LPS induced a reduction in the expression of IRAK1 and an increase in P-TAK1. The LPS-induced decreases in the levels of IRAK1 and the LPS-induced phosphorylation of P-TAK1 were suppressed in a berteroin dose-dependent manner (Figure 5A). The levels of P-IKKα/β and P-IκBα increased markedly by LPS but berteroin pretreatment suppressed these changes. However, the levels of IKKα and IKKβ did not change due to treatment with either LPS or berteroin. LPS treatment markedly reduced the levels of IκBα but berteroin effectively suppressed this decrease (Figure 5B). Actually, 9 μmol/L berteroin almost completely prevented the LPS-induced phosphorylation of IKKα/β and IκBα and the LPS-induced degradation of IκBα. LPS increased the levels of P-AKT, P-p38 MAPK, and P-ERK1/2 in Raw 264.7 cells and pre-treatment with berteroin significantly prevented the increases. The levels of AKT, p38 MAPK, and ERK1/2 were not altered by either LPS or berteroin treatment (Figure 5C).

### 2.4. Berteroin Inhibits TPA-Induced Ear Edema Formation in Mice

**Figure 6:**
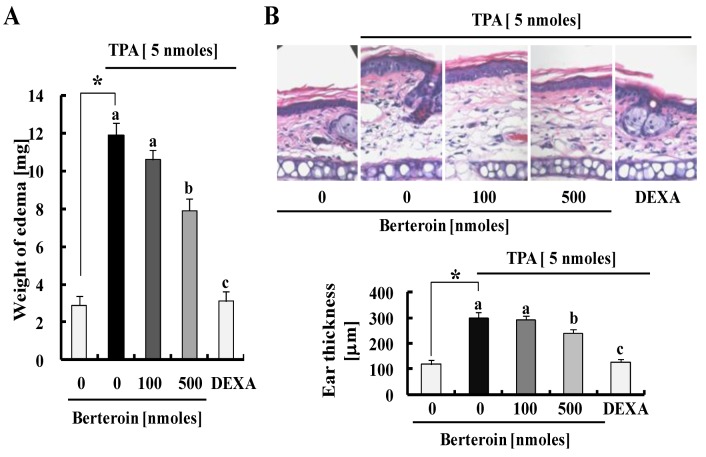

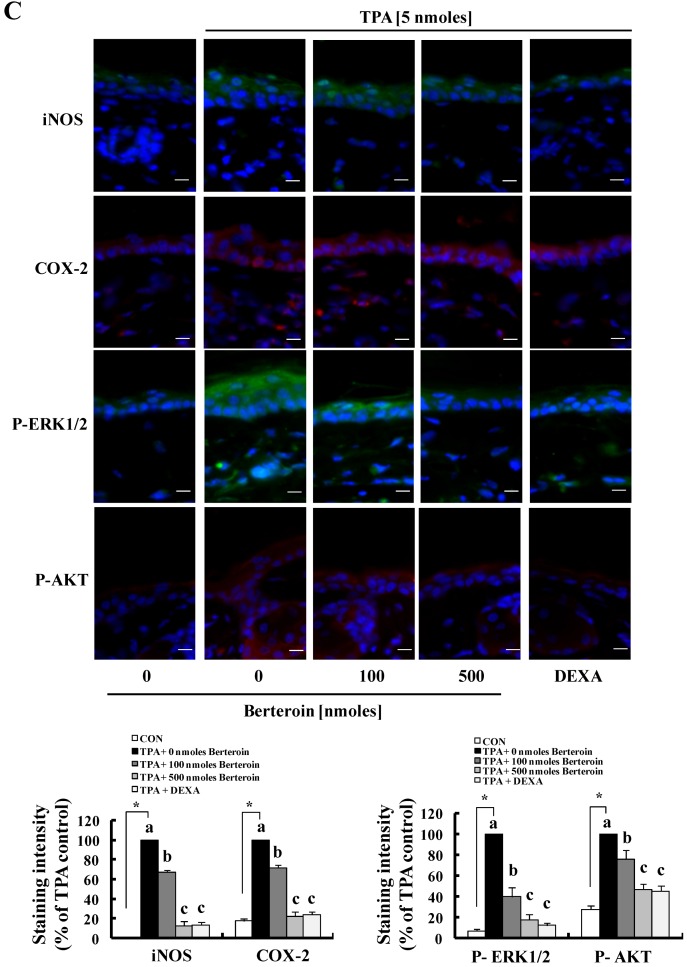

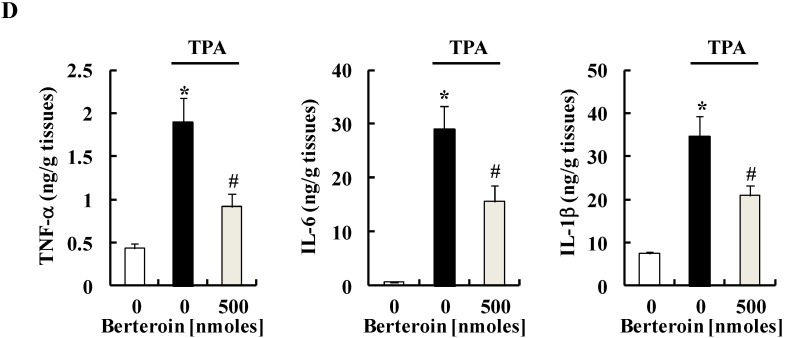
Berteroin inhibits 12-O-tetradecanoylphorbol-13-acetate (TPA)-induced ear edema formation in mice. ICR mouse ears were treated with berteroin (0, 100 or 500 nmole/ear) or dexamethasone (50 μg/ear) prior to topically applying TPA. The mice were sacrificed after a 4 h TPA treatment. (**A**) Changes in ear weights were measured in 6-mm diameter ear punch samples; (**B**) Each mouse ear section was stained with hematoxylin and eosin (H&E) and representative H&E images are shown in the upper panel. Ear thickness of each sample stained with H&E was quantified (lower panel). Each bar represents mean ± SEM (*n* = 10); (**C**) Expression levels of inducible nitric oxide synthase (iNOS), cyclooxygenase (COX)-2, phosphorylated extracellular regulated kinase (P-ERK)1/2, and P-AKT in the ear sections of each mouse were evaluated by immunofluorescence staining (upper panel). Scale bar, 10 μm. Relative expression levels of iNOS, COX-2, P-ERK1/2, and P-AKT were compared (lower panel). Each bar represents mean ± SEM (*n* = 6). * Significantly different from the vehicle-treated control group (*p* < 0.05). Means with different letter (a, b or c) are significantly different among the berteroin groups (*p* < 0.05). DEXA, dexamethasone; (**D**) Tissue lysates were prepared from ear punch samples and the concentrations of TNF-α, IL-6, and IL-1β in mouse skin were measured using the relevant ELISA kits. Each bar represents mean ± SEM (*n* = 6). * Significantly different from the vehicle-treated control group (*p* < 0.05). ^#^ Significantly different from the TPA group (1 mg/L TPA + 0 μmol/L berteroin) (*p* < 0.05).

We used a TPA-induced mouse ear edema model to validate the anti-inflammatory efficacy of berteroin observed *in vitro*. After treatment with various concentrations of berteroin or vehicle in left ears of ICR mice, we induced ear edema by topically applying TPA. TPA induced a remarkable increase in ear weights, and this increase was effectively suppressed by pre-treatment with 500 nmoles berteroin (Figure 6A). These changes in ear weight due to TPA and berteroin were accompanied by similar changes in ear thickness determined by hematoxylin and eosin staining of ear tissues (Figure 6B). Additionally, immunofluorescence (IF) staining of ear tissues showed that TPA increased expression of iNOS and COX-2 as well as the levels of P-ERK1/2 and P-AKT, and berteroin pre-treatment (100 nmoles) significantly suppressed the TPA-induced changes (Figure 6C). Dexamethasone (DEXA, 50 μg/ear) also effectively inhibited the TPA-induced changes in ear weights and thickness as well as the levels of iNOS, COX-2, P-ERK1/2, and P-AKT (Figure 6A–C). Furthermore, ELISA results showed that berteroin pre-treatment significantly inhibited TPA-induced production of TNF-α, IL-6, and IL-1β in mouse skin (Figure 6D).

### 2.5. Discussion

The anti-inflammatory properties of ITCs present in cruciferous vegetables including erucin [24], sulforaphane [27], BITC [23], and PITC [22] have been reported previously. In the present study we demonstrated that the sulforaphane analogue berteroin exhibited powerful anti-inflammatory properties using both *in vitro* and *in vivo* experimental models. Berteroin at a dose of 500 nmoles in ICR mice effectively inhibited TPA-stimulated ear edema formation as well as the expression of iNOS and COX-2 at a dose of 100 nmoles. Additionally, berteroin effectively inhibited LPS-induced production of NO, PGE_2_, TNF-α, IL-6, and IL-1β in murine macrophages. These results indicate that berteroin is a good candidate anti-inflammatory agent.

Macrophages play a critical role activating the inflammatory process through the production of various cytokines and growth factors (reviewed in [28]). Among the growth factors and cytokines produced by macrophages, we demonstrated that berteroin potently inhibited mRNA expression and secretion of the pro-inflammatory cytokines TNF-α, IL-6, and IL-1β (Figure 2). This result indicates that the inhibition of these cytokines contributed to inflammation inhibition by berteroin. In addition to these cytokines, our results clearly show that berteroin inhibited expression of iNOS and COX-2 by blocking gene transcription of these enzymes (Figure 1B–D). The decrease in enzyme levels is probably the most important reason for the reduction in the levels of NO and PGE_2_ in berteroin-treated macrophages (Figure 1B). In addition to *in vitro* results, we observed that TPA-induced inflammation was accompanied by the expression of COX-2 and iNOS (Figure 6C) and the production of TNF-α, IL-6, and IL-1β (Figure 6D) in mouse skin. Additionally, berteroin potently inhibited the expression of the enzymes and the production of the cytokines. Because it has been shown that infiltrated macrophages express COX-2 proteins and secrete pro-inflammatory cytokines (especially IL-6) [29,30], our *in vivo* results suggest that berteroin suppresses TPA-induced inflammatory responses via the inhibition of pro-inflammatory cytokine production and COX-2 expression in infiltrated macrophages.

Macrophages can be broadly classified as M1 and M2 macrophages. Classically activated M1 macrophages induced by LPS or IFN-γ express the inflammation-related enzymes (iNOS and COX-2) and pro-inflammatory cytokines (TNF-α, IL-6, and IL-1β). However, alternatively activated M2 macrophages induced by IL-6, IL-13, or IL10 play an anti-inflammatory role [31]. As expected, LPS treatment increased the expression of iNOS and COX-2 and the secretion of TNF-α, IL-6, and IL-1β, which were suppressed by berteroin treatment (Figure 1 and Figure 2). In addition to these M1 mediators, berteroin inhibited LPS-induced gene expression of CD80 and CD86 (other M1 markers) with no changes in that of MMR (a M2 marker) (Figure 3). A recent study showed that macrophages with the M1 or M2 phenotype can be re-polarized to the M2 or M1 phenotype by IL-4 or LPS/IFN-γ, respectively [32]. Our results indicate that berteroin exerts anti-inflammatory properties via inhibiting the expression of pro-inflammatory mediators and the differentiation into M1 macrophages.

IL-1, TNF, COX-2, and iNOS expression is largely regulated by the NF-κB signaling pathway [12,33]. Several reports have shown that PITC, BITC, erucin, and allyl isothiocyanate exhibit anti-inflammatory properties by obstructing NF-κB signaling in macrophages, resulting in the inhibition of pro-inflammatory enzyme and cytokine expression [22,23,24,34]. Consistent with these results, we clearly showed that berteroin inhibited NF-κB signaling by decreasing the DNA binding activity of its p65 subunit in Raw 264.7 cells (Figure 4). The anti-inflammatory effects of berteroin resulted from inhibiting IKKα/β phosphorylation and the subsequent phosphorylation of IκBα leading to the inhibition of IκBα degradation (Figure 5B). We further found that LPS-induced degradation of IRAK1 and phosphorylation of TAK1 were markedly inhibited in a berterion concentration-dependent manner without changes in the levels of TLR4, MyD88, or TAK1 (Figure 5A). These results indicate that reducing P-TAK1 contributed to the reduction in IKK phosphorylation in murine macrophages. Future studies are needed to determine how berteroin inhibits IRAK1 degradation and TAK1 phosphorylation.

AKT, ERK1/2, and p38 MAPK also regulate the production of pro-inflammatory mediators and cytokines and NF-κB is a target of these signaling molecules [25,35,36]. AKT stimulates the transactivation potential of the RelA/p65 subunit of NF-κB by utilizing of IKK and activating p38 MAPK [37]. Phosphorylation of AKT, p38 MAPK, and ERK1/2 induced by LPS stimulation was substantially inhibited in Raw 264.7 cells after treatment with berteroin (Figure 5C). Additionally, the levels of P-ERK1/2 and P-AKT decreased significantly by 100 nmoles berteroin in TPA-treated mouse skin (Figure 6C). These results indicate that down-regulating AKT and MAPK signaling by berteroin may have contributed to the inhibition of the NF-κB signaling pathway, which subsequently led to the reduction in the expression of pro-inflammatory cytokines and enzymes.

Several studies have examined the mechanisms by which various ITCs exert anti-inflammatory effects. Consistent with the present observation, one of the mechanisms underlying the anti-inflammatory effects of ITCs is the inhibition of the NF-κB pathway [22,23,24,38]. It has also been reported that the anti-inflammatory effects of sulforaphane, a structurally similar isothiocyanate, were associated with decreased levels of oxidative stress and increased levels of antioxidant enzyme (heme oxygenase 1) as well as the inhibition of DNA binding of various transcription factors (CCAAT/enhancer binding protein, cAMP response element-binding protein, and activator protein 1) [39,40]. Unfortunately, the anti-oxidative properties of berteroin have not been reported. As more pathways other than NF-κB may also play roles in the anti-inflammatory effect of berteroin, further studies are needed to explore anti-inflammatory mechanisms of berteroin.

Unlike sulforaphane and erucin (other sulforaphane analogs), the concentrations of berteroin in cruciferous vegetables and the bioavailability of berteroin have not been well studied. Jirovetz *et al.*, reported the aroma compounds released from rocket salad (*Eruca sativa*) using headspace solid phase microextraction (SPME). In this study, the concentrations of berteroin (as the main compound) were 9.3% of the aroma compounds (calculated as % peak area of gas chromatography analysis using a nonpolar column) [20]. Although berteroin is expected to exist in cruciferous vegetables as considerable concentrations, further studies are needed to determine the concentrations of berteroin in cruciferous vegetables.

## 3. Experimental Section

### 3.1. Cell Culture and Secretion of Cytokines and pro-Inflammatory Mediators by Raw 264.7 Cells

Raw 264.7 cells (American Type Culture Collection, Manassas, VA, USA) were maintained in DMEM containing 10% FBS, 100,000 U/L penicillin, and 100 mg/L streptomycin.

To examine the anti-inflammatory effects of berteroin, Raw 264.7 cells were serum-deprived for 16 h in DMEM containing 10 mL/L FBS, then treated with 0, 3, 6, or 9 μmol/L berteroin (Santa Cruz Biotechnology, Santa Cruz, CA, USA) and/or LPS (1 mg/L) for 24 h, and culture media were harvested. NO concentrations were measured using the Griess reagent system (Promega, Madison, WI, USA). PGE_2_, TNF-α, IL-6, and IL-1β concentrations were measured using ELISA kits: PGE_2_, TNF-α, and IL-6 (R&D Systems, Minneapolis, MN, USA); and IL-1β (eBioscience, San Diego, CA, USA). Cell viability was measured by the MTT [3-(4,5-dimethylthiazol-2-yl)-2,5-diphenyltetrazolium bromide] method.

### 3.2. mRNA Levels of pro-Inflammatory Mediators and Cytokines in Raw 264.7 Cells

Serum-deprived Raw 264.7 cells were incubated with berteroin and/or LPS for 6 h. Total RNA was extracted using the RNeasy Plus Mini Kit (Qiagen, Valencia, CA, USA) and reverse-transcribed using the Maxime^TM^ RT PreMix (iNtRON Biotechnology, Seongnam, Korea). Real-time PCR was conducted as described previously [41]. The real-time PCR results were normalized to GAPDH.

### 3.3. Reporter Gene Assays of pro-Inflammatory Enzymes

Raw 264.7 cells were transiently co-transfected with plasmids harboring control vector (pRL-TK) and iNOS (pGL-miNOS-1588 [42]) or COX-2 (pGL-mCOX-2-724 [43]) (Promega) using Nucleofector-II (Amaxa, Gaithersburg, MD, USA). After transfection, the cells were plated in DMEM supplemented with 100 mL/L 10% FBS. The cell monolayers were serum-deprived and incubated with berteroin and/or LPS for 6 h. Luciferase activity of cell lysates was measured using Dual Luciferase Assay System (Promega). The values of luciferase activity in each construct were normalized with those of control pRL-TK activity.

### 3.4. Western Blot Analysis

Western blot analyses were performed using total cell lysates, cytosolic fractions, or nuclear fractions as described previously [41]. The following antibodies were used: iNOS and COX-2 (BD Transduction Laboratories, Palo Alto, CA, USA); TLR4, MyD88, IRAK1, NFκB p65, α-tubulin or lamin B (Santa Cruz Biotechnology), P-TAK1, TAK1, IκBα, P-IκBα (Ser32), IKKα, IKKβ, P-IKKα (Ser180)/IKKβ (Ser181), ERK-1/2, P-ERK-1/2, AKT, P-AKT, p38 MAPK and P-p38 MAPK (Cell Signaling Technology Inc., Beverly, MA, USA). β-actin, α-tubulin, or lamin B was used as an internal control. Densities of signals were estimated using Image J software (NIH, Bethesda, MD, USA).

### 3.5. Electrophoretic Mobility Shift Assay (EMSA)

Serum-deprived Raw 264.7 cells (2 × 10^6^ cells/dish) were pretreated with berteroin for 40 min followed by an additional 20-min stimulation with LPS (1 mg/L). The cells were harvested and nuclear extracts were isolated [41]. EMSA was performed described as previously [41]. In brief, nuclear extracts were incubated with a [γ-^32^P]-labeled NF-κB DNA probe for 30 min and then subjected to a 5% non-denaturing gel, and DNA binding of NF-κB was visualized by autoradiography.

### 3.6. TPA-Induced Mouse Ear Edema

After acclimatization to laboratory conditions, 4-week-old female ICR mice (Orient Bio Inc., Gapyung, Korea) were divided into five groups (10 mice/group): vehicle [dimethyl sulfoxide (DMSO)/acetone], TPA (5 nmoles), TPA/berteroin (100 nmoles), TPA/berteroin (500 nmoles) and TPA/dexamethasone (DEXA, 50 μg). TPA and berteroin were dissolved in DMSO/acetone (*v/v*, 15/85). The left ears of mice were topically treated with vehicle, berteroin or DEXA for 1 h, and additionally treated with TPA for 4 h. After the TPA treatment, mice were sacrificed, and a 6 mm diameter disc from each ear was removed with a metal punch and weighed. The edema from ear biopsies was calculated by subtracting the weight of the right ear (non-treated) from that of the left ear (treated). To investigate whether berteroin inhibits the production of pro-inflammatory cytokines in TPA-induced mouse ear edema model, total tissue lysates were prepared as previously described [23] and utilized in ELISA as described above. All animal experimental protocols were approved by the Animal Care and Use Committee of Hallym University (Hallym2011-04).

### 3.7. Immunohistochemical and Immunofluorescence (IF) Staining

Paraffin-embedded sections (5 μm) of ear biopsies were stained with hematoxylin and eosin. IF stainings were conducted with antibodies against iNOS, COX-2 (Cayman Chemicals, Ann Arbor, MI, USA), P-AKT, or P-ERK1/2 as described previously [24]. Nuclei were counterstained with 4',6-diamidino-2-phenylindole. Images were captured using Carl Zeiss AxioImager microscope (Jena, Germany), and fluorescent intensities were quantified with Image J software.

### 3.8. Statistical Analyses

Results are expressed as means ± SEM. Differences between the control group and the LPS (or TPA) group were assessed via the Student’s *t*-test. Then, ANOVA tests were performed to determine whether berteroin has significant effects on LPS-treated cells (or TPA-treated mice). When the ANOVA results indicated differences among the four groups (for example, LPS + 0 μmol/L berteroin; LPS + 3 μmol/L berteroin; LPS + 6 μmol/L berteroin; LPS + 9 μmol/L berteroin), Duncan’s multiple range tests were conducted to find which two groups are different. Different letters indicate statistically significant differences between the four groups. All statistical operations were performed using SAS software for Windows, ver. 9.2 (SAS Institute, Cary, NC, USA). Differences were considered significant at *p* < 0.05.

## 4. Conclusions

**Figure 7:**
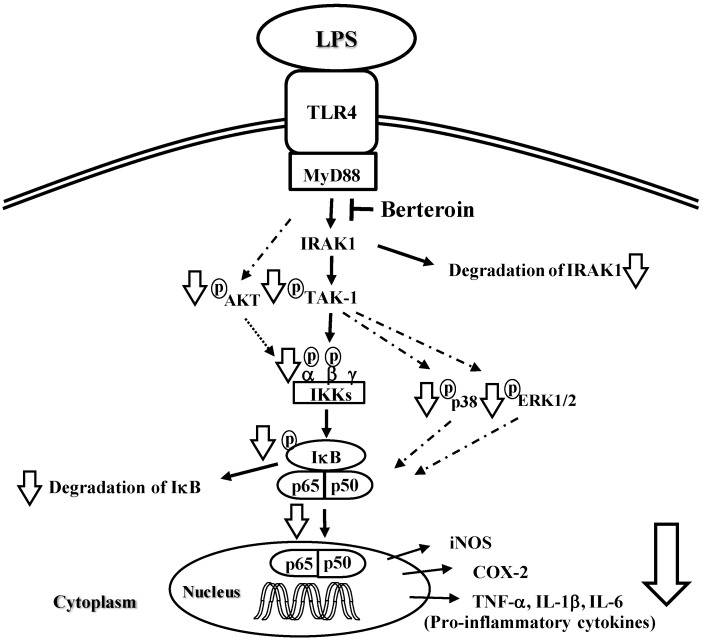
Proposed mechanisms underlying the anti-inflammatory effects of berteroin. Berteroin inhibits IL-1 receptor-associated kinase (IRAK1) degradation, which results in decreased phosphorylation of transforming growth factor β activated kinase-1 (TAK1) leading to the reduction of IκB kinase (IKK), p38 mitogen activated protein kinase (MAPK), and extracellular regulated kinase (ERK)1/2 phosphorylation and subsequent reduction of IκB phosphorylation and degradation. Decreased AKT phosphorylation also contributes to the reduction of IKK activation. The resulting high levels of IκB prevents p65 translocation from the cytosol to the nucleus thereby preventing transcription of inducible metric oxide synthase (iNOS), cyclooxygenase (COX)-2, tumor necrosis factor (TNF)-α, interleukin (IL)-1β, and IL-6 leading to a decreased inflammatory response.

We demonstrated that berteroin effectively inhibited LPS-stimulated inflammatory responses in murine macrophages as well as TPA-stimulated skin edema formation in mice. Berteroin prevented LPS-induced phosphorylation of TAK1, AKT, p38 MAPK, and ERK1/2, IKKα/β, and IκBα as well as the degradation of IRAK1 and IκBα in murine macrophages. The increased IκBα bound to NF-κB and thereby prevented the translocation of this transcription factor to the nucleus. As a result, berteroin inhibited expression of iNOS, COX-2, IL-1β, IL-6, and TNF-β (Figure 7). The finding that only 500 nmoles of berteroin effectively inhibited skin inflammation in mice suggests that berteroin is a good candidate as an anti-inflammatory agent.
